# Editorial: Community series in early life stress and depression, Volume II

**DOI:** 10.3389/fpsyt.2025.1408329

**Published:** 2025-02-17

**Authors:** Fushun Wang, Jiongjiong Yang, Fang Pan, James A. Bourgeois, Jason H. Huang

**Affiliations:** ^1^ Department of Psychology, Sichuan Normal University, Chengdu, Sichuan, China; ^2^ School of Psychological and Cognitive Sciences and Beijing Key Laboratory of Behavior and Mental Health, Peking University, Beijing, China; ^3^ Department of Psychology, Shandong University, Jinan, Shandong, China; ^4^ Department of Psychiatry, University of California Davis Medical Center, Sacramento, CA, United States; ^5^ Department of Neurosurgery, Baylor Scott & White Health, Temple, TX, United States

**Keywords:** basic emotion, MDD, major depressive disorder, stress, early life stress

Major depressive disorder (MDD) is one of the most common psychiatric disorders, significantly burdening modern society. It is estimated that MDD seriously affects the quality of life for more than 20% of the global population, ranking it among the most prevalent health problems (1). Despite of the significant progress in understanding the pathology and treatment of MDD, its etiology remains complex. The monoamine hypothesis, proposed in the 1950s, suggested that deficiencies in monoamine neurotransmission (including norepinephrine, dopamine, and serotonin) may cause MDD. Even though the current first-line antidepressant treatments are still targeting monoamine neurotransmitters, there are many debates about the role of monoamines on MDD (2, 3). Thus, many recent studies propose alternative hypotheses for MDD, including early life stress induced neurotrophic neurogenesis, inflammation, oxidative stress, and gut bacteria (4).

## Early life stress

Stress, particularly early life stress, is a critical factor in MDD. Numerous studies have shown that adverse childhood experiences increase susceptibility to depression and that depression with such experiences has specific clinical-biological features (5). Freud suggested that early life stress is stored in the subconscious and leads to neurosis in adulthood (6). Bowlby and others suggested that early life stress induces coping styles or behavioral changes, known as attachment styles (7). Despite extensive research into the mechanisms linking stress and affective disorders, the precise mechanisms remain elusive. Many recent studies suggested that early life stress induced neurotrophic neurogenesis, inflammation, oxidative stress, and gut bacteria might be the leading causes for MDD (8). Among them, the neuroimmune interplay and epigenetic regulation are the most prevalent topic on the etiology of MDD. For example, some recent studies have found that early life stress induced epigenetic changes might be a major risk factor for suicide in MDD (9).

## Epigenetic changes

Early life stress can induce epigenetic changes in monoamine neurotransmitters and hormone receptors (10), as well as neuroimmune system, potentially contributing to MDD. Recent epigenetic studies have explored how early life adversity impacts long-term mental and physical health (11). Epigenetic mechanisms can encode different early life events through varying genome expression (12). For instance, some early life events have been shown to induce epigenetic changes in neuromodulator receptors, such as DNA methylation of ACTH receptors or monoamine oxidase (MAO) (13). Alterations in the HPA axis via epigenetic modifications have also been observed in both humans and animals, leading to changes in receptor expression and dysfunction of the HPA axis and monoamines (14). In addition, early life stress might also induce neurotrophic neurogenesis, inflammation and oxidative stress (15).

In conclusion, early life stress has attracted significant research efforts to understand the mechanisms underlying affective disorders. It has provided a clear link between early life stress and increased susceptibility to these disorders. A comprehensive understanding of the mechanisms involved in stress is essential for the clinical management of affective diseases. With this in mind, we set out this Research Topic, similar to the one in 2020, to welcome advanced studies on the mechanisms—from social community factors to molecular factors—involved in early life stress and their relationship with affective disorders.

First, in an interesting experimental paper “5hmC Modification Regulates R-loop Accumulation in Response to Stress,” Xu et al. studied R-loops, which are three-stranded structures formed by nascent mRNA with the DNA template strand. These R-loop structures form a barrier to transcription and DNA replication. The study found that chronic restraint stress increased R-loop accumulation and decreased 5hmC modification in the prefrontal cortex (PFC) of stressed mice. Administration of the antidepressant fluoxetine effectively decreased R-loop accumulation and DNA damage. The study suggested that epigenetic DNA 5hmC modification negatively regulates R-loop accumulation under stress. This study provides potential therapeutic targets for depression.

Second, Jirakran et al. studied the relationship between neuroticism and MDD, and suggested that neuroticism, a personality trait, can predict major depressive disorder (MDD). The research article was titled “The Effects of Adverse Childhood Experiences on Depression and Suicidal Behaviors are Partially Mediated by Neuroticism, a Forme Fruste of Major Depression”. They found that early life neglect (physical and emotional) and abuse (physical, emotional, and sexual) were partially mediated by neuroticism in inducing MDD. The authors concluded that neuroticism is a much better predictor of MDD than extra-version and agreeableness, while openness and conscientiousness do not have any significant effect.

Third, another paper titled “Prevalence and Associated Clinical Factors for Overweight and Obesity in Young First-Episode and Drug- Naive Chinese Patients with Major Depressive Disorder,” Zhang et al. studied the clinical factors of obesity/overweight in young first-episode MDD patients. They found that being overweight are common in young patients with major depressive disorder (MDD), and might be a predictors for MDD. In addition, they reported that the high incidence of overweight and obesity in young MDD patients were related with serum TSH which might be identified as a common risk factor. Thus, the authors suggested that key population information (age, age of onset, and gender) can be screened in clinical practice to guide weight control and improve the prognosis of MDD.

Recently, many studies have found that the gut microbiota can influence MDD through the “microbe-gut-brain axis” and that the composition and function of the gut microbiota are influenced by early stress. In the review paper titled “Adverse Childhood Experience and Depression: The Role of Gut Microbiota,” Bai et al. directly linked adverse childhood experiences, gut microbiota with MDD. This article reviewed recent advances on these relationships and found that gut microbiota may regulate the development of MDD through the neuroendocrine pathway, which involves the HPA axis, monoamine neurotransmitters, neurotrophins, and metabolites in neuroendocrine processes.

In summary, these papers collectively contribute to our understanding of how early life stress affects affective disorders, highlighting both molecular genetic changes and personality effects on MDD. The integration of traditional theories, such as the monoamine hypothesis, with new findings on mechanisms such as neurotrophic neurogenesis, inflammation, oxidative stress, the hypothalamic-pituitary-adrenal (HPA) axis, synaptic plasticity, and gut flora, offers a more comprehensive understanding of MDD. A comprehensive understanding of these mechanisms is essential for the clinical management of affective diseases. Early identification and intervention strategies targeting these pathways could significantly improve outcomes for individuals with MDD. As research continues to evolve, it is crucial to explore and validate these new hypotheses and treatments, aiming to alleviate the burden of MDD on individuals and society.
